# S13-1: The experience of parkrun by those with mental health conditions: results from the parkrun UK Health and Wellbeing Survey 2018

**DOI:** 10.1093/eurpub/ckae114.255

**Published:** 2024-09-26

**Authors:** Helen Quirk, Alice Bullas, Steve Haake, Suzy Ker

**Affiliations:** Sheffield Hallam University, Sheffield, England; Sheffield Hallam University, Sheffield, England; Sheffield Hallam University, Sheffield, England; Tees, Esk and Wear Valleys NHS Foundation Trust

## Abstract

**Purpose:**

Physical activity can alleviate symptoms of mental health conditions such as depression and anxiety and has been recognised as an effective treatment option, but suitable activities are often lacking. parkrun is a free, weekly 5km walk/run and volunteer initiative. The 2018 parkrun UK Health and Wellbeing Survey identified participants who self-reported mental health conditions. The aim was to understand motives for taking part, the impact of participation and the characteristics of parkrun that influence their experience.

**Methods:**

Findings from 1,574 respondents were analysed using mixed methods. Quantitative data were analysed using chi square and logistic regression to explore the reported motives and perceived impact of parkrun. Open text responses about the impact of parkrun on health and wellbeing were analysed using thematic analysis.

**Results:**

Health conditions included anxiety disorder (n = 902), depression (n = 1,202), panic attacks (n = 225), and Post-Traumatic Stress Disorder (n = 150). This group was significantly more likely than those with no health conditions to choose mental health, condition management and happiness as motives for participation in parkrun. They were more likely to report that parkrun improved their mental health and confidence compared to those with no health conditions. Qualitative responses revealed that people benefitted from the regular, social, outdoors, and volunteering aspects of parkrun. For many people, parkrun was deemed inclusive and catalysed other positive experiences such as improved social connections, running ability and health choices. It was praised for being accessible due to the mixed abilities and option to walk or volunteer. However, parkrun was “not all sunshine and roses” with some reports of social anxiety, concerns about being too slow, coming last and not fitting in.

**Conclusions:**

Inclusive physical activity provision in the community is often lacking for people with mental health conditions. This study has found a wide range of benefits of parkrun, particularly for people who are socially isolated and less active. For sustained participation, it is crucial that new participants are supported to overcome anxieties and feel included.

**Funding:**

None

